# Intestinal Macrophages Balance Inflammatory Expression Profiles via Vitamin A and Dectin-1-Mediated Signaling

**DOI:** 10.3389/fimmu.2020.00551

**Published:** 2020-03-31

**Authors:** Martje N. Erkelens, Gera Goverse, Tanja Konijn, Rosalie Molenaar, Marieke R. Beijer, Jan Van den Bossche, Kyra E. de Goede, Sanne G. S. Verberk, Wouter J. de Jonge, Joke M. M. den Haan, Reina E. Mebius

**Affiliations:** ^1^Department of Molecular Cell Biology and Immunology, Amsterdam Infection and Immunity Institute, Amsterdam UMC, Vrije Universiteit Amsterdam, Amsterdam, Netherlands; ^2^Tytgat Institute for Gastro Intestinal and Liver Research, Amsterdam Gastroenterology Endocrinology and Metabolism Research Institute, Amsterdam UMC, University of Amsterdam, Amsterdam, Netherlands

**Keywords:** macrophage, retinoic acid, Dectin-1, pro-inflammatory, anti-inflammatory, vitamin A, small intestine

## Abstract

Tissue resident intestinal macrophages are known to exhibit an anti-inflammatory phenotype and produce little pro-inflammatory cytokines upon TLR ligation, allowing symbiotic co-existence with the intestinal microbiota. However, upon acute events such as epithelial damage and concomitant influx of microbes, these macrophages must be able to quickly mount a pro-inflammatory response while more inflammatory macrophages are recruited from the blood stream simultaneously. Here, we show that dietary intake of vitamin A is required for the maintenance of the anti-inflammatory state of tissue resident intestinal macrophages. Interestingly, these anti-inflammatory macrophages were characterized by high levels of Dectin-1 expression. We show that Dectin-1 expression is enhanced by the vitamin A metabolite retinoic acid and our data suggests that Dectin-1 triggering might provide a switch to induce a rapid production of pro-inflammatory cytokines. In addition, Dectin-1 stimulation resulted in an altered metabolic profile which is linked to a pro-inflammatory response. Together, our data suggests that presence of vitamin A in the small intestine enhances an anti-inflammatory phenotype as well as Dectin-1 expression by macrophages and that this anti-inflammatory phenotype can rapidly convert toward a pro-inflammatory state upon Dectin-1 signaling.

## Introduction

Macrophages are mostly tissue-resident mononuclear phagocytes that have a tissue-specific role besides immune surveillance and clearing of pathogens. Interestingly, macrophages are plastic cells that can either have pro- or anti-inflammatory properties. Nevertheless, it is not clear how individual macrophages switch between these phenotypes (1). To address their inflammatory versus regenerative properties, macrophages are often characterized as either pro-inflammatory M1 macrophages or anti-inflammatory M2 macrophages, which are two ends of a broad spectrum (2). While tissue-resident macrophages are often anti-inflammatory macrophages, pro-inflammatory macrophages dominate the early stages of inflammation, and it is thought that macrophage polarization happens under the influence of environmental cues such as pathogen-associated molecular patterns and cytokines (3, 4).

*In vitro*, type one stimuli such as interferon (IFN) γ and lipopolysaccharide (LPS) lead to the development of murine M1 macrophages or classically activated macrophages whereas type two stimuli such as interleukin (IL)-4 induce murine M2 macrophages or alternatively activated macrophages (5). Pro-inflammatory M1 macrophages are characterized by their production of nitric oxide (NO) and the production of pro-inflammatory cytokines such as IL-12, IL-6, and TNFα. Conversely, anti-inflammatory M2 macrophages are characterized by high expression of Arginase-1 and anti-inflammatory cytokines such as IL-10 (6). Besides the differences in inflammatory capacity, it has also been reported that pro- and anti-inflammatory macrophages use different metabolic pathways to meet their energy demand (7). Whereas anti-inflammatory macrophages use oxidative phosphorylation for ATP production, pro-inflammatory macrophages rely on glycolysis for the rapid production of ATP and biosynthetic intermediates necessary for the production of pro-inflammatory cytokines and reactive oxygen species (8, 9). Overall, macrophages within the tissues can have a broad spectrum of functions, of which the pro-inflammatory M1 and anti-inflammatory M2 classification is an artificial representation of this range of functionalities.

The largest pool of macrophages resides in the intestines (10). Here, they form a first line of defense for invading pathogens. In addition, gut-resident macrophages have an important role in the maintenance of immune homeostasis and produce little pro-inflammatory cytokines upon TLR ligation (11). Therefore, they are capable of clearing invading microorganisms without causing inflammation. In most tissues, tissue-resident macrophages derive from the yolk sac during embryonic development and do not re-circulate via the blood (12). However, although embryonic progenitors are able to maintain themselves in the intestine, most of the tissue-resident macrophages in lamina propria of the intestines are continuously replenished by blood monocytes (13, 14). When these monocytes enter the intestine, they express high levels of Ly6C and intermediate levels of the chemokine receptor CX3CR1, which upon maturation into resident intestinal macrophages changes toward low Ly6C expression and high CX3CR1 expression (15). These CX3CR1^high^ tissue-resident macrophage are described as anti-inflammatory macrophages that play a role in maintaining tissue homeostasis (16, 17). Interestingly, during inflammation, CX3CR1 expression remains low and the monocytes entering from the bloodstream appear to differentiate toward macrophages with a pro-inflammatory profile (15, 18).

Because aberrant inflammation in the gut can result in severe pathologies such as inflammatory bowel disease or celiac disease, determining the factors that regulate the differentiation of entering monocytes might aid the development of therapies for these diseases. Previous studies indicated that during intestinal inflammation, incoming monocytes develop a pro-inflammatory phenotype under the influence of pathogen recognition receptor signaling (15, 18). On the other side of the spectrum, the vitamin A metabolite retinoic acid, readily available in the intestine and required for maintaining intestinal immune tolerance, was shown to induce an anti-inflammatory phenotype in murine macrophages (19, 20). Overall, one can state that the intestinal micro-environment determines the phenotypic properties of monocytes immediately upon their entry into the gut but the exact mechanisms of macrophage polarization in the small intestine need further study (21).

Here, we aimed to study the mechanisms by which intestinal monocyte differentiation is regulated in further detail. We found that retinoic acid indeed induced a more anti-inflammatory phenotype both *in vivo* as well as *in vitro*. Interestingly, anti-inflammatory macrophages expressed high levels of the C-type lectin-like receptor Dectin-1, which was further enhanced by retinoic acid. Stimulation with the Dectin-1 ligand curdlan appeared to rapidly overthrow the anti-inflammatory state of these macrophages by skewing them toward a pro-inflammatory phenotype (22). The expression of Dectin-1 on anti-inflammatory macrophages might act as a safety measure allowing macrophages to switch to a pro-inflammatory state, for example when the intestinal barrier breaks. In conclusion, our data demonstrate that retinoic acid enhances an anti-inflammatory phenotype in macrophages and that this anti-inflammatory role can be reversed by Dectin-1 stimulation. Expression of Dectin-1 by intestinal macrophages thus creates a mechanism enabling a rapid switch from an anti- to a pro-inflammatory phenotype.

## Materials and Methods

### Mice

C57BL/6 mice (Charles River Laboratories, Maastricht, Netherlands), were mated overnight and the morning of vaginal plug detection was marked as E0.5. Starting at E8-E9, pregnant females either received a chemically defined diet that lacked vitamin A (the modified AIN-93M feed; MP Biomedicals, Santa Ana, CA, United States) or contained vitamin A (4,000 IU/kg in the modified AIN-93M feed; MP Biomedicals, Santa Ana, CA, United States) with use of vitamin-free casein. All mice were housed under specific pathogen-free conditions. Pups were weaned at 4 weeks of age and maintained on the same diet until at least 10 weeks of age, when analyses were performed. For the generation of retinoic acid high mice, C57BL/6 adult mice (12–14 weeks of age) were fed with global 16% protein rodent diet (Harlan, Horst, Netherlands) which contained 4.5 μg retinoic acid per gram dry food supplemented with 100 μg retinoic acid per gram dry food or vehicle control for 1–2 weeks (Sigma-Aldrich, Zwijndrecht, Netherlands). Diet was refreshed twice a day to reduce the effects of RA degradation by light. All animal experiments were approved by the Ethical Committee of the VU University Medical Center.

### Preparation of Small Intestine Cell Suspension

Small intestines were dissected and opened longitudinally after removal of Peyer’s patches. Small intestines were washed with HBSS without Ca^2+^ and Mg^2+^ (Invitrogen, Landsmeer, Netherlands) containing 15 mM HEPES (Invitrogen, Landsmeer, Netherlands) and 250 μg/ml gentamicin (Invitrogen, Landsmeer, Netherlands) to remove luminal contents. Small intestinal segments were incubated twice with HBSS containing 5 mM EDTA (Sigma-Aldrich, Zwijndrecht, Netherlands), 15 mM HEPES (Invitrogen, Landsmeer, Netherlands), 10% FCS (HyClone Laboratories/Greiner Bio-One, Alphen aan den Rijn, Netherlands), 1 μM DTT (Promega Benelux, Leiden, Netherlands), and 14 mM β-ME (Sigma-Aldrich, Zwijndrecht, Netherlands) for 15 min at 37°C while constantly stirring. Pieces of small intestine were minced and digested at 37°C for 20 min, using 150 μg/ml Liberase Blendzyme 2 (Roche, Penzberg, Germany) and 200 μg/ml DNAse I (Roche, Almere, Netherlands) in HBSS containing 15 mM HEPES and 10% FCS while constantly stirring. The cell suspensions were filtered through 70-μm cell strainers (BD Biosciences, Breda, Netherlands) and the recovered cells were washed twice with HBSS without Ca^2+^ and Mg^2+^ containing 15 mM HEPES. CD45^+^ cells were positively selected using PE-Cy7–labeled anti-CD45 (clone 30-F11; eBioscience/Immunosource, Halle-Zoersel, Belgium) and the EasySep PE positive selection kit (StemCell Technologies, Grenoble, France). Purified CD45^+^ lamina propria cells were used as cell suspensions for flow cytometry and analyzed with a Cyan ADP flow cytometer (Beckman Coulter, Woerden, Netherlands).

### Immunofluorescence

Tissues were embedded and frozen in OCT compound (Sakura Finetek Europe, Zoeterwoude, Netherlands), 7 μm cryosections were generated, fixed in acetone for 5 min and air-dried for 10 min. Sections were then pre-incubated with PBS supplemented with 10% (v/v) mouse serum for 15 min. Incubation with the primary antibody for 30 min was followed by incubation with secondary antibodies which were conjugated with Alexa fluorophores (Invitrogen Life Technologies, Breda, Netherlands) for 30 min together with directly labeled antibodies that did not cross react. All incubations were carried out at room temperature. Sections were analyzed on a Leica DM6000 Microscope (Leica Microsystems Nederland BV, Rijswijk, Netherlands).

### Antibodies

Anti-CD11c-PE, anti-CD11b-Biotin (both eBioscience, San Diego, United States), and anti-F4/80 (clone CI:A3-1) and anti-Dectin-1 (clone 2A11), which were affinity-purified from hybridoma cell culture supernatants with protein G-Sepharose (Pharmacia, Uppsala, Sweden), were used for immunofluorescence. For FACS analysis anti-CD11c-Pacific Blue, anti-CD11b-APC, anti-CD11b-PE, anti-CD45-PE-Cy7, anti-F4/80-PE-Cy5 (all eBioscience, San Diego, United States), anti-Dectin-1-FITC (Serotec Oxford, United Kingdom) and unlabeled anti-CX3CR1 (Abcam, Cambridge, United Kingdom) were used. Live/Dead Fixable Near-IR stain fluorescence (Invitrogen, Landsmeer, Netherlands) was used to exclude dead cells.

### *In vitro* Experiments

Bone marrow was obtained by flushing femurs and tibia from wild type C57BL/6 mice using 1 ml culture media DMEM (Life Technologies, Grand Island, NY, United States) containing 10% FCS, 1% L-glutamine, and 1% penicillin-streptomycin (BioWhittaker Europe, Verviers, Belgium). Cells were washed with culture media after which they were cultured for 1 week in culture media containing 15% conditioned medium from macrophage-colony stimulating factor secreting L929 fibroblasts at 37°C. After 1 week, the cells were exposed either to 100 ng/ml IL-4 (ImmunoTools, Friesoythe, Germany) or with 10 ng/ml Escherichia coli LPS (Sigma Aldrich) and 5 × 10^3^ U/ml IFN-γ (U-CyTech, Utrecht, Netherlands) for 48 h. These stimulations were done in the presence or absence of 100 nM retinoic acid and when indicated, cells were subsequently exposed to a specific ligand for Dectin-1 (10 μg/ml Curdlan; Sigma Aldrich, Zwijndrecht, Netherlands) for an additional 24 h. Subsequently, cells were taken up in Trizol (Invitrogen, Landsmeer, Netherlands) for mRNA isolation, while supernatants were collected for ELISA.

### Quantitative Real-Time PCR

RNA was isolated from Trizol samples by precipitation with isopropanol and chloroform according to manufacturer’s protocol. cDNA was synthesized from total RNA using RevertAid First Strand cDNA Synthesis Kit (Fermentas Life science, Leon-Rot, Germany) according to manufacturer’s protocol and qPCR was performed using SYBR Green mastermix (Foster City, CA, United States) on StepOne real-time PCR systems from Applied Biosystems (Bleiswijk, Netherlands). Used primer sequences are listed in Table 1. The genes cyclophylin and ubiquitin were used as reference genes.

**TABLE 1:** Primer sequences used for RT-qPCR.

**Gene**	**Forward**	**Reverse**
*Ubq*	AGCCCAGTGTTACCACCAAG	ACCCAAGAACAAGCACAAGG
*Cyclo*	ACCCATCAAACCATTCCTTCTGTA	TGAGGAAAATATGGAACCCAAAGA
*Il10*	GGACAACATACTGCTAACCG	GGGGCATCACTTCTACCAG
*Il12*	AGACCCTGCCCATTGAACTG	CGGGTCTGGTTTGATGATGTC
*Il6*	GAGTTGTGCAATGGCAATTCTG	TGGTAGCATCCATCATTTCTTTGT
*Arg1*	GCTCTGGGAATCTGCATGGGC	TGGCAGATATGCAGGGAGTCACC
*Tnfa*	TGGAACTGGCAGAAGAGGCACT	CCATAGAACTGATGAGAGGGAGGC
*CLEC7A (Dectin-1)*	TTGAGTTCATTGAAAGCCAAACA	CAGAACCATGGCCCTTCACT

### Enzyme-Linked Immunosorbent Assay (ELISA)

Supernatants of cell cultures were analyzed for IL-6, IL-12 p70, TNFα, and IL-10 using respective uncoated ELISA kits (Invitrogen, Landsmeer, Netherlands) according to manufacturer’s protocol. Samples were analyzed using a microplate reader (Biorad, Veenendaal, Netherlands).

### Nitric Oxide Assay

Griess reagent consisting of 2.5% H_3_PO_4_, 1% sulphanilamide and 0.1% naphtylene diamine dihydrochloride (all Sigma Aldrich, Zwijdrecht, Netherlands) in miliQ water was added to supernatants 1:1. Samples were analyzed using a microplate reader (Biorad, Veenendaal, Netherlands).

### Metabolic Extracellular Flux Analysis

Metabolic flux analysis was performed as detailed earlier (23). Briefly, bone marrow-derived macrophages were cultured in XF-96-cell culture plates at a concentration of 50,000 cells per well (Agilent, Santa Clara, CA, United States). Macrophages were skewed with either IL-4 or LPS and IFNγ or remained untreated naïve macrophages. Oxygen consumption (OCR) rate and extracellular acidification rate (ECAR) were measured in an XF-96 Flux Analyzer (Seahorse analyzer, Agilent, Santa Clara, CA, United States). Changes in OCR and ECAR in response to glucose (first injection), oligomycin (second injection), FCCP, and pyruvate (third injection) and antimycin A and rotenone (fourth injection) were used to calculate all oxidative phosphorylation and glycolysis parameters as detailed in Supplementary Figure S2.

### Statistics

Results are given as mean ± SEM. Statistical analysis were performed using GraphPad Prism 8 (La Jolla, CA, United States) with either the 2-tailed Student’s *t* test or Two-Way ANOVA with Bonferroni’s correction, significance is indicated by ^∗^ significance if *p* < 0.05, ^∗∗^significance if *p* < 0.01 or ^∗∗∗^ significance if *p* < 0.005.

## Results

### Vitamin A Deficient Mice Show a More Pro-inflammatory Type of Macrophages in the Small Intestine

**FIGURE 1:**
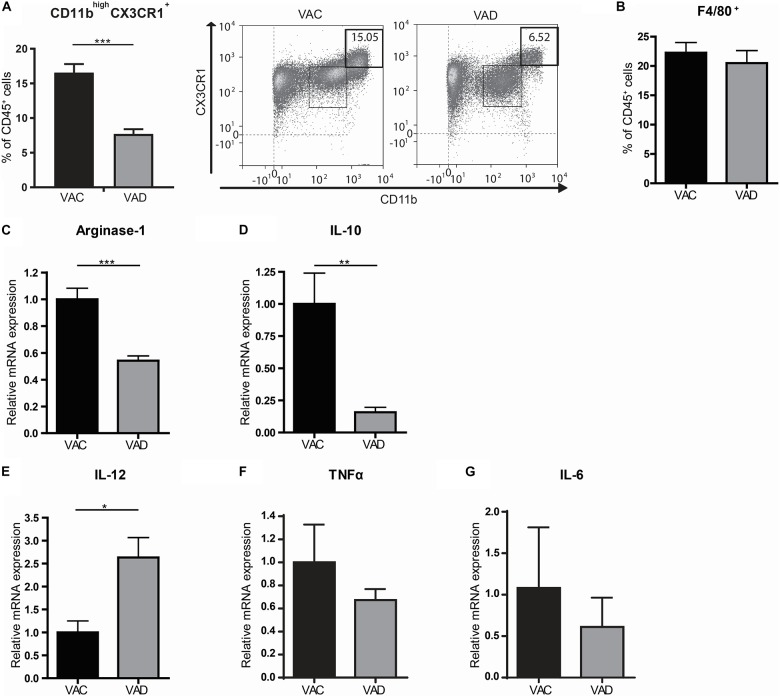
The small intestines of vitamin A deficient mice have a more pro-inflammatory macrophage phenotype. Mice were either raised on a vitamin A deficient (VAD) or a vitamin A conventional (VAC) diet. The small intestine was removed, made into a single cell suspension and CD45^+^ cells were analyzed by flow cytometry for their proportion of CD11b and CX3CR1 **(A)** and F4/80 **(B)**. Additional gating strategies can be found in Supplementary Figure S1. In addition, small intestines of both VAD and VAC mice were analyzed by qPCR for the expression of Arginase-1 **(C)**, IL-10 **(D)**, IL-12 **(E)**, TNFα **(F)**, and IL-6 **(G)**. Expression was normalized to the reference genes Cyclophylin and Ubiquitin. Relative expression to the control group was shown. Significant differences are indicated by *p < 0.05, **p < 0.01, or ***p < 0.005 (*n* = 10, ±SEM).

In order to determine the role of retinoic acid for the homeostatic control of macrophages, we used vitamin A deficient (VAD) mice. These mice are completely depleted from vitamin A and thus unable to produce retinoic acid. Interestingly, we observed a decrease in CD11b^high^CX3CR1^+^CD45^+^ cells, which include anti-inflammatory macrophages, in the small intestines of VAD mice compared to vitamin A competent (VAC) mice while the proportion of F4/80^+^ CD45^+^ cells, which includes macrophages but also monocytes, eosinophils and some dendritic cell subsets, remained unaffected (Figures 1A,B). In addition, we compared the gene expression of the anti-inflammatory macrophage markers Arginase-1 and IL-10 and the pro-inflammatory macrophage markers IL-12β, TNFα, and IL-6 in the small intestine of VAD and VAC mice. Interestingly, we observed that the expression of Arginase-1 and IL-10 was reduced in VAD mice compared to VAC mice while the expression of IL-12 was enhanced in VAD mice (Figures 1C–E). However, expression levels of TNFα and IL-6 were unaffected in VAD mice (Figures 1F,G). These results suggested that dietary intake of vitamin A is required for maintenance of the resident phenotype and tissue homeostasis.

### Retinoic Acid Reduces the Pro-inflammatory Phenotype in Macrophages *in vivo* and *in vitro*

**FIGURE 2:**
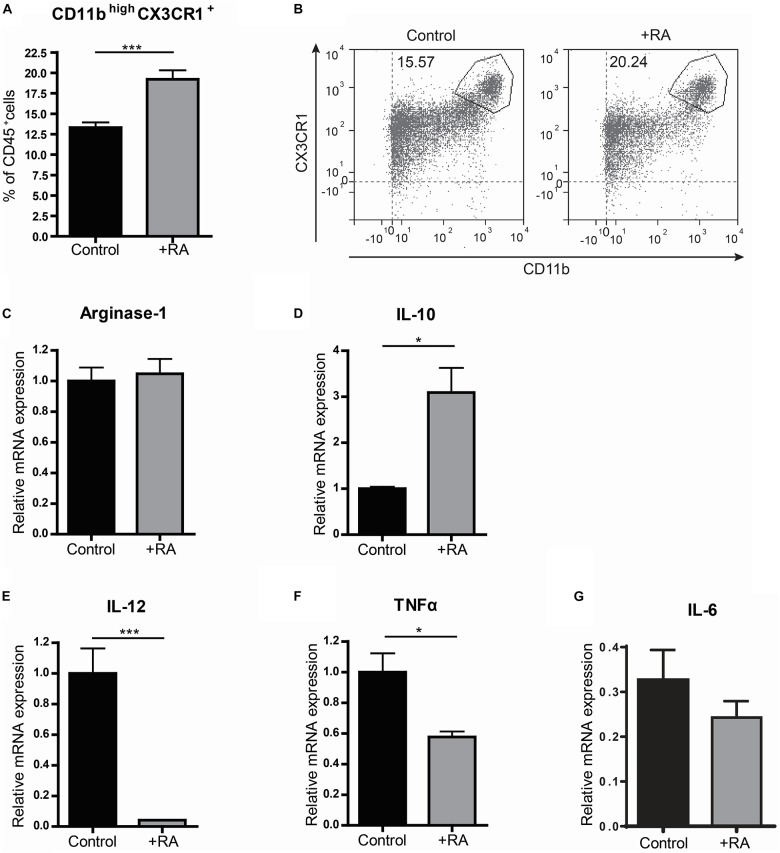
Retinoic acid reduces pro-inflammatory properties in macrophages *in vivo*. Mice were either raised on a normal control diet (control) or a diet supplemented with retinoic acid (+RA). The small intestine was removed, made into a single cell suspension and CD45^+^ cells were analyzed by flow cytometry for their proportion of CD11b and CX3CR1 **(A,B)**. In addition, small intestines of both control mice and mice that were fed with a diet complemented with retinoic acid were analyzed by qPCR for the expression of Arginase-1 **(C)**, IL-10 **(D)**, IL-12 **(E)**, TNFα **(F)**, and IL-6 (**G**). Significant differences are indicated by *p < 0.05, or ***p < 0.005 (*n* = 5–10, ±SEM).

**FIGURE 3:**
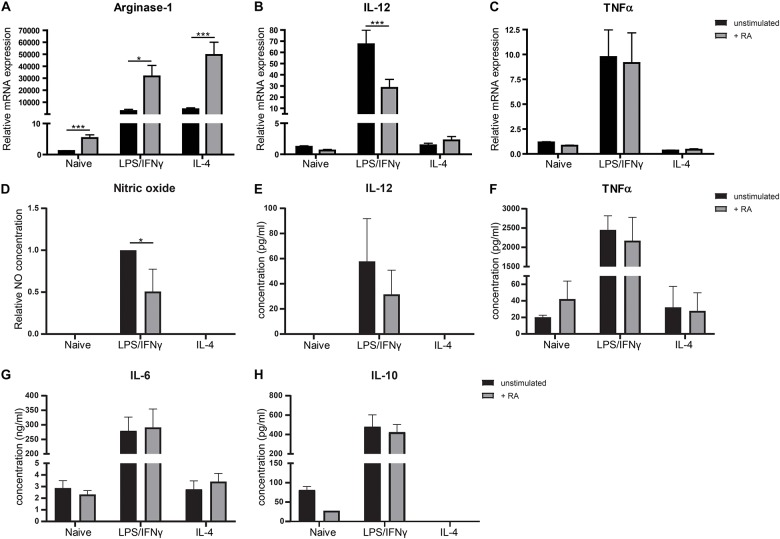
Retinoic acid reduces pro-inflammatory properties in macrophages *in vitro*. Bone-marrow derived macrophages stimulated with LPS and IFNγ or IL-4 or naïve macrophages cultured with (in black) or without (in gray) the presence of retinoic acid for 48 h were analyzed for the expression of Arginase-1 **(A)**, IL-12 **(B)**, and TNFα **(C)**. Expression was normalized to the reference genes Cyclophylin and Ubiquitin. Concentrations of nitric oxide **(D)**, IL-12 **(E)**, TNFα **(F)**, IL-6 **(G)**, and IL-10 **(H)** were determined in supernatants of cell cultures. Data for nitric oxide was normalized to the unstimulated samples. Concentrations of NO and IL-12 did not exceed detection limit in naïve and IL-4 treated cells. IL-10 concentrations could not be quantified in IL-4 treated macrophages. Significant differences are indicated by *p < 0.05, **p < 0.01, or ***p < 0.005 (*n* = 3–10, ±SEM).

Because VAD mice showed to have pro-inflammatory type macrophages in the small intestine, we questioned whether addition of retinoic acid to the diet could have the opposite effect and would result in more anti-inflammatory macrophages. Addition of retinoic acid to the diet for 2 weeks resulted in more CD11b^high^CX3CR1^+^ CD45^+^ cells, which include anti-inflammatory macrophages, when compared to mice fed with the control diet (Figures 2A,B). In addition, we observed a reduced gene expression of the pro-inflammatory markers IL-12 and TNFα in the small intestine of mice that received retinoic acid-supplemented diet, while the expression of IL-10, associated with anti-inflammatory macrophages, was increased (Figures 2D–F). However, no differences in the expression of Arginase-1 and IL-6 between these animals were observed (Figures 2C,G). To further substantiate our data, we added retinoic acid to naïve, pro-, and anti-inflammatory skewed bone-marrow derived macrophages in an *in vitro* model and we observed an increased gene expression of Arginase-1 compared to control cells (Figure 3A). In addition, we observed a reduction in the expression of IL-12 in pro-inflammatory skewed macrophages after addition of retinoic acid to the culture (Figure 3B), while this did not impact the expression of TNFα (Figure 3C). In addition, we analyzed the supernatants of the cell cultures for NO concentration and the presence of IL-12, TNFα, IL-6 and IL-10 (Figures 3D–H). Corresponding with the increased expression of Arginase-1, which can inhibit the production of NO, we observed reduced levels of NO in supernatants derived from LPS/IFNγ skewed macrophages treated with retinoic acid (Figure 3D). However, retinoic acid treatment did not alter the production of IL-12, TNFα, IL-6 or IL-10 (Figures 3E–H). Nevertheless, our data suggest a role for retinoic acid in maintaining a resident anergic state in macrophages.

### Retinoic Acid Enhances the Expression of Dectin-1

**FIGURE 4:**
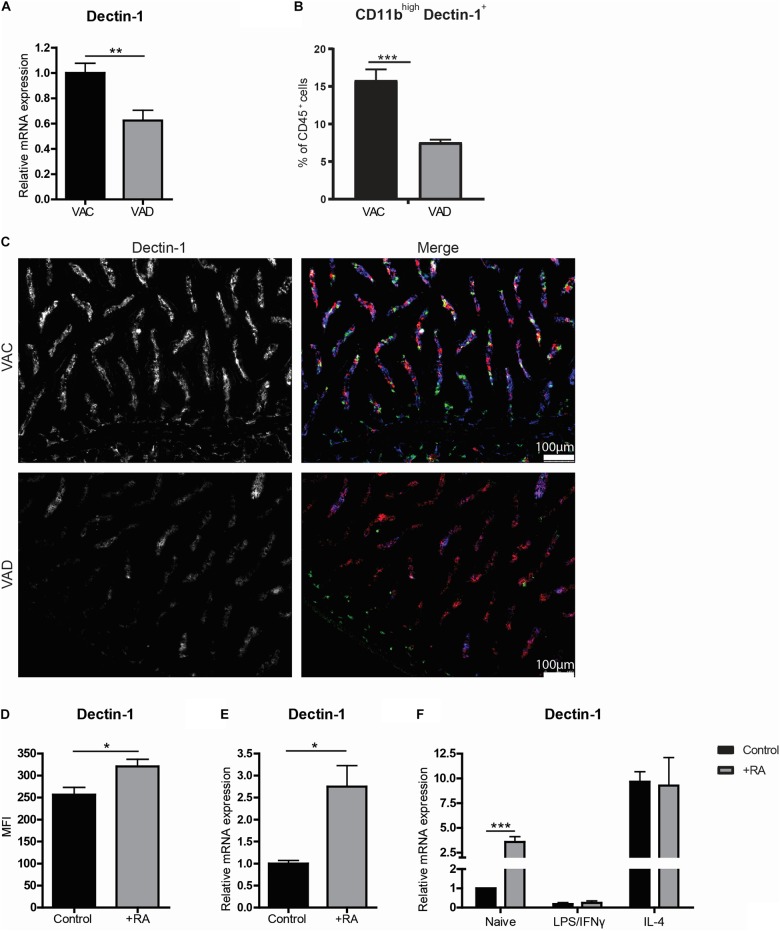
Anti-inflammatory macrophages express high levels of Dectin-1. Mice were either raised on a vitamin A deficient (VAD) or a vitamin A conventional (VAC) diet. Small intestine was removed and mRNA was extracted or the tissue was made into a single cell suspension or the tissue was embedded for analysis by fluorescent microscopy. **(A)** qPCR analysis of the small intestine of VAC and VAD mice for the expression of Dectin-1. **(B)** CD45 + cells derived from the single cell suspension from the small intestines of VAC and VAD mice were analyzed by flow cytometry for their proportion of CD11b and Dectin-1. **(C)** Microscopy images of the small intestine of VAC and VAD mice were made after staining with Dectin-1 (blue), CD11b (green) and CD11c (red). **(D,E)** In a different setting, the expression of Dectin-1 was analyzed by flow cytometry in CD45^+^ cells derived from the small intestine of mice that were fed with a diet with or without the addition of retinoic acid **(D)** and the expression of Dectin-1 in the small intestine was determined by qPCR **(E)**. Bone-marrow derived macrophages stimulated with LPS and IFNγ or IL-4 or naïve macrophages cultured with (in black) or without (in gray) retinoic acid were analyzed for the expression of Dectin-1 **(F)**. Expression was normalized to the reference genes Cyclophylin and Ubiquitin. Relative expression to the control group was shown. Significant differences are indicated by *p < 0.05, or ***p < 0.005 (*n* = 5–10, ±SEM).

Interestingly, we observed an overall reduced expression of the β-glucan receptor Dectin-1 in the small intestines of VAD mice using RT-qPCR (Figure 4A). This reduction was confirmed to be specific for CD11b^+^ macrophages, as determined by flow cytometry (Figure 4B). Using microscopy, this reduction of Dectin-1 was confirmed to be restricted to CD11b^+^CD11c^–^ cells present in the lamina propria of mice on vitamin A sufficient diet, and strongly reduced in the lamina propria of mice fed a vitamin A-deficient diet (Figure 4C). Moreover, dietary intake of retinoic acid further induced the expression of Dectin-1 compared to mice fed with a control diet (Figures 4D,E). To check whether retinoic acid can directly induce Dectin-1 in macrophages, retinoic acid was added to the *in vitro* cultures. In these cultures, retinoic acid induced Dectin-1 only in naïve macrophages while the addition of retinoic acid did not affect the expression of Dectin-1 in macrophages treated with LPS and INFγ or IL-4 (Figure 4F). Remarkably, the expression of Dectin-1 was distinctly higher in IL-4-treated macrophages regardless of the addition of retinoic acid (Figure 4F). These data suggest that retinoic acid plays a role in the induction of Dectin-1 expression in naïve macrophages and that Dectin-1 is highly expressed by anti-inflammatory macrophages both *in vivo* and *in vitro*.

### Dectin-1 Ligation Enhances a Pro-inflammatory Phenotype in IL-4-Treated Macrophages

**FIGURE 5:**
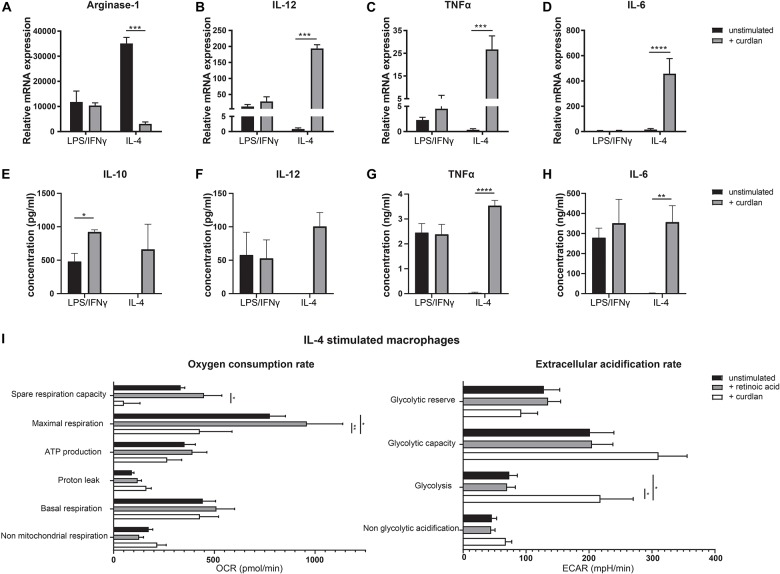
Dectin-1 stimulation results in a more pro-inflammatory phenotype. Bone marrow derived macrophages were cultured and skewed toward a pro-inflammatory phenotype using LPS and IFNγ or toward an anti-inflammatory phenotype using IL-4, with or without the addition of retinoic acid for 24 h. Subsequently, macrophages were stimulated with the Dectin-1 ligand curdlan for an additional 24 h **(A–D)**. Relative expression of Arginase-1 **(A)**, IL-12 **(B)**, TNFα **(C)**, and IL-6 **(D)** determined by qPCR. Expression was normalized to the reference genes Cyclophylin and Ubiquitin. **(E–H)** Concentration of IL-10 **(E)**, IL-12 **(F)**, TNFα **(G)**, and IL-6 **(H)** in the supernatants of cell cultures of IL-4 stimulated or LPS/IFNγ stimulated macrophages after stimulation with or without curdlan determined by ELISA. IL-10 and IL-12 concentrations did not exceed detection limit in IL-4 treated macrophages without curdlan stimulation. **(I)** Oxygen consumption rate (OCR) and extracellular acidification rate (ECAR) of IL-4 stimulated macrophages after stimulation with retinoic acid or curdlan. Various metabolic parameters were determined as described in Supplementary Figure S3. Changes in OCR and ECAR over time are represented in Supplementary Figure S4. Significant differences are indicated by *p < 0.05, **p < 0.01, ***p < 0.005, or ****p < 0.001 (*n* = 3, ±SEM).

As Dectin-1 is highly expressed by anti-inflammatory macrophages, we hypothesized that this might act as a mechanism to overthrow the anti-inflammatory phenotype in case of an acute infection for example upon barrier breach. To study the functional effects of Dectin-1 signaling in these cells, we stimulated bone marrow derived macrophages, untreated or skewed with either LPS and IFNγ or IL-4, with the Dectin-1 ligand curdlan for 24 h. Then, we analyzed the expression levels of Arginase-1, IL-12, TNFα and IL-6 (Figures 5A–D). As expected, stimulation with curdlan did not affect LPS/IFNγ treated macrophages. However, the expression of Arginase-1 was greatly reduced upon treatment with curdlan in IL-4 treated macrophages (Figure 5A). At the same time, the expression of IL-12, TNFα and IL-6 was significantly elevated in IL-4 treated macrophages upon Dectin-1 stimulation (Figures 5B–D). In addition, we observed an increased production of IL-10, TNFα and IL-6 in the supernatants of cultured IL-4 treated macrophages and untreated macrophages as a result of Dectin-1 triggering while IL-12 production was increased in IL-4 treated macrophages but not in naïve macrophages (Figures 5E–H and Supplementary Figure S2). As pro- and anti-inflammatory macrophages are described to be metabolically distinct, we questioned whether Dectin-1 ligation impacted the metabolic status of IL-4 treated macrophages. Therefore, we performed extracellular flux analysis to measure the oxygen consumption rate (OCR) as a measurement of mitochondrial oxidative phosphorylation, and the extracellular acidification rate (ECAR) as a measurement of glycolysis. We observed that retinoic acid stimulation does not affect the metabolic state of IL-4 stimulated macrophages. Conversely, Dectin-1 stimulation with curdlan, induced a reduction in oxidative phosphorylation and an increase in glycolysis in IL-4-stimulated macrophages, a shift to a metabolic state that is characteristic of pro-inflammatory macrophages (Figure 5I) (24). Together, these observations indicate that Dectin-1 ligation induces both a phenotypic and metabolic shift toward a more pro-inflammatory profile in macrophages.

## Discussion

The role of retinoic acid in maintaining immune homeostasis has been recognized for more than 100 years but is yet to be completely understood (25). Here, we show that retinoic acid plays a role in the induction of anti-inflammatory properties in macrophages in the small intestine. We observed a reduction of CD45^+^CD11b^high^CX3CR1^+^ anti-inflammatory macrophages in the small intestine of VAD mice while the total amount of CD45^+^F4/80^+^ macrophages was unaffected. In addition, qPCR analysis of the small intestine of VAD mice revealed a reduced expression of the anti-inflammatory macrophage markers Arginase-1 and IL-10 but an increased expression of the pro-inflammatory cytokine IL-12 compared to VAC mice. Overall, these observations indicate that although the total amount of macrophages is unaffected, the inflammatory signature of macrophages in the small intestine of VAD mice has shifted toward a more pro-inflammatory profile; suggesting a regulatory role for retinoic acid in intestinal macrophages as also been reported in previous studies (19, 20). In addition, a previous study has shown that vitamin A deficiency aggravates colitis in a DSS model whereas vitamin A supplementation ameliorated colitis in mice (26, 27). Vitamin A thus has an important role in the maintenance of anti-inflammatory responses in the intestine and our data now show that the intestinal macrophages are likely to also participate in these pro- and anti-inflammatory responses.

Moreover, a possible regulatory role for retinoic acid in intestinal macrophages was supported by the observation that the addition of retinoic acid to the diet of mice resulted in an induction of CD45^+^CD11b^high^CX3CR1^+^ anti-inflammatory macrophages and reduced expression of the pro-inflammatory cytokines IL-12 and TNFα but induced expression of IL-10 in the small intestine; which is in line with previously reported *in vitro* and *in vivo* studies in both mouse and human (28, 29). *In vitro* we showed that bone marrow derived macrophages skewed toward a pro-inflammatory phenotype using LPS and IFNγ expressed higher levels of Arginase-1, resulting in a reduced production of NO upon treatment with retinoic acid. In addition, retinoic acid reduced the expression of IL-12 in LPS/IFNγ treated macrophages although we did not observe a difference in IL-12 production upon retinoic acid treatment. Overall, these observations suggest that retinoic acid can impact macrophages phenotypically and functionally by enhancing an anti-inflammatory response in the small intestine.

Interestingly, we observed that macrophages with anti-inflammatory properties have high expression levels of the β-glucan receptor Dectin-1. Moreover, we observed a reduction in Dectin-1 expression levels in macrophages in the small intestine of VAD animals whereas increased Dectin-1 expression was observed in the small intestines of mice fed with a retinoic acid supplemented diet. Furthermore, addition of retinoic acid to *in vitro* cultured naïve bone marrow derived macrophages induced Dectin-1 expression levels. These observations suggest that retinoic acid might play a role in the induction of Dectin-1 expression in macrophages. However, when we stimulated naïve macrophages with retinoic acid in the presence of the pan-retinoic acid receptor agonist BMS, we were not able to block the induction of Dectin-1, suggesting that the effect of retinoic acid was independent of retinoic acid receptor binding (data not shown). Interestingly, a previous study showed that retinoic acid reduced the expression of Dectin-1 in human monocytes during a *Candida Albicans* infection but that this effect was independent of retinoic acid receptor signaling (30). As it was shown that retinoic acid can have a differential effect on macrophages depending on the presence or absence of an immune challenge, it could be that the effect of retinoic acid on Dectin-1 expression level depends on the inflammatory context (31). Together, these observations suggest that retinoic acid is involved in enhancing the expression levels of Dectin-1 in macrophages under homeostatic conditions but that this is not directly depending on retinoic acid receptor mediated signaling.

In addition, Dectin-1 stimulation *in vitro* resulted in an increased production of the cytokines IL-10, IL-12, TNFα and IL-6 in Dectin-1 high expressing IL-4 stimulated macrophages, suggesting a switch toward a pro-inflammatory state. Furthermore, Dectin-1 ligation resulted in a metabolic shift from oxidative phosphorylation toward glycolysis in IL-4 stimulated macrophages, which is also characteristic of a pro-inflammatory response (24). And thus, retinoic acid appeared to enhance an anti-inflammatory phenotype while it did not affect pro-inflammatory (data not shown) or Dectin-1 stimulated macrophages. However, it should be noted that these experiments were performed in bone-marrow derived macrophages as an artificial model. LPS/IFNγ treated bone-marrow derived macrophages have stronger cytokine responses than intestinal tissue resident macrophages due to the LPS stimulation. Furthermore, IL-4 signaling is mediated via Stat6, which has been shown to reduce IL-10 expression and might explain why we observed less IL-10 production in IL-4 treated macrophages than in naïve macrophages upon Dectin-1 triggering (32). Nevertheless, we observed that retinoic acid can reduce both nitric oxide production and IL-12 expression in IL-4 treated macrophages. Together, these observations translate to a hypothesis that the expression of Dectin-1 might act as a safety switch to quickly induce a pro-inflammatory profile in macrophages in the gut in acute situations such as barrier breach.

This mechanism might be important because intestinal macrophages are less responsive toward TLR signaling (15, 33, 34). However, unlike TLRs, Dectin-1 is capable of discriminating between soluble and particulate ligands. This means that Dectin-1 is differentially activated depending on the size of the ligand and it was shown that Dectin-1 activation by whole glucan particles (such as curdlan) induces a stronger inflammatory response (35). This differential downstream signaling via Dectin-1 enables macrophages to distinguish the presence of microbes from microbe-derived peptides. Because intestinal macrophages line the epithelium and sample the intestinal lumen, anergy toward soluble microbial ligands prevents an unnecessary inflammatory response. The high expression of Dectin-1 however, will enable intestinal macrophages to respond quickly upon an influx of intact microbes, ensuring adequate protection in acute situations. Together, these interpretations suggest that Dectin-1 expression is important for tissue-resident intestinal macrophages and that retinoic acid regulates Dectin-1 expression levels on incoming monocytes during homeostasis, providing a safety switch in case of an acute situation. In human, Dectin-1 deficiency results in impaired cytokine responses upon fungal infection and recurring mucocutaneous infections (36). It has therefore been hypothesized that Dectin-1 deficiency leads to fungal dysbiosis. Interestingly, it has been shown that fungal dysbiosis in the intestine results in elevated susceptibility to intestinal inflammation and has been associated with inflammatory disease (37, 38). Therefore, retinoic acid might prevent the development of fungal dysbiosis by enhancing Dectin-1 expression in intestinal macrophages and therewith provide another mechanism to reduce the risk of developing intestinal inflammation.

Here, we show that retinoic acid enhances an anti-inflammatory phenotype in the small intestine of mice. In addition, we observed that anti-inflammatory macrophages express high levels of the β glucan receptor Dectin-1 and that this expression is enhanced by retinoic acid under homeostatic conditions. As we observed that Dectin-1 ligation induced a pro-inflammatory response, our data suggest that the high expression of Dectin-1 on anti-inflammatory macrophages in the gut might act as a mechanism to ensure a rapid response toward microbes upon barrier breach. This might play an important role in preventing the development of fungal dysbiosis. Together, our data provides valuable insights in the mechanisms behind the maintenance of immune homeostasis in the small intestine.

## Data Availability Statement

The datasets generated for this study are available on request to the corresponding author.

## Ethics Statement

The animal study was reviewed and approved by the VU University Medical Center.

## Author Contributions

ME, GG, TK, RM, and MB performed the experiments. ME, GG, JB, KG, SV, and RM designed the experiments. ME and GG analyzed the data. GG, WJ, JH, and RM conceptualized the project. ME, GG, and REM wrote the manuscript. RM obtained funding for the project.

## Conflict of Interest

The authors declare that the research was conducted in the absence of any commercial or financial relationships that could be construed as a potential conflict of interest.

## Supplementary Material

FIGURE S1 Gating strategy Small intestines were removed and made into a single cell suspension. **(A)** Live CD45^+^ cells were analyzed for CX3CR1 and CD11b expression and a gate for CD11b^high^CX3CR1^+^ macrophages was set. **(B)** Gating strategy to determine proportion of F4/80^+^CD45^+^ cells of all lamina propria cells in vitamin A competent (VAC) and vitamin A deficient (VAD) mice.

FIGURE S2 Effects of Dectin-1 stimulation in naïve macrophages. Bone marrow derived macrophages were cultured for 24 h with or without retinoic acid and were stimulated with the Dectin-1 ligand curdlan for an additional 24 h **(A–D)**. Concentration of IL-10 **(A)**, IL-12 **(B)**, TNFα **(C)**, and IL-6 **(D)** in the supernatants of cell cultures of IL-4 stimulated or LPS/IFNγ stimulated macrophages after stimulation with or without curdlan determined by ELISA. **(E)** Oxygen consumption rate (OCR) and extracellular acidification rate (ECAR) of naïve macrophages after stimulation with retinoic acid or curdlan. Various metabolic parameters were determined as described in Supplementary Figure S3. Changes in OCR and ECAR over time are represented in Supplementary Figure S4. Significant differences are indicated by ^∗^*p* < 0.05, ^∗∗^*p* < 0.01, ^∗∗∗^*p* < 0.005, or ^****^*p* < 0.001, (*n* = 3, ±SEM).

FIGURE S3 Analysis method extracellular flux assay. **(A)** When analyzing the oxygen consumption rates (OCR in pMoles/min), oligomycin injection allows to calculate the oxygen consumption used for mitochondrial ATP synthesis. Carbonyl cyanide 4-(trifluoromethoxy)phenylhydrazone (FCCP) uncouples mitochondrial respiration and the corresponding OCR measurements yield data about the maximal and spare respiratory capacity. Finally, injection of rotenone (Rot) and antimycin A (AA) block mitochondrial complex I and III and the residual OCR represents the non-mitochondrial oxygen consumption. **(B)** After the injection of glucose, the increase in extracellular acidification rate (ECAR in mpH/min) represents the glycolysis rate. The additional increase in ECAR after ATP synthase inhibition with oligomycin (OM) provides information about the glycolytic reserve and capacity. After normalization for cell numbers using protein concentration, following metabolic parameters are calculated as follows:Basal respiration = OCR (6) – OCR (15)Proton leak = OCR (9) – OCR (15). ATP production = OCR (6) – OCR (9). Maximum respiration = OCR (12) – OCR (15). Spare respiratory capacity = OCR (12) – OCR (6). Non-glycolytic acidification = ECAR (3). Glycolysis = ECAR (6) – ECAR (3). Maximum glycolytic capacity = ECAR (9) – ECAR (3). Glycolytic reserve = ECAR (9) – ECAR (6). This figure and legend were adapted from a previously published report (24).

FIGURE S4 Changes in oxygen consumption rate and extracellular acidification rate of naive and IL-4 stimulated macrophages over time. Bone marrow derived macrophages were cultured and kept unstimulated or were skewed toward an anti-inflammatory phenotype using IL-4. Subsequently, macrophages were stimulated with the Dectin-1 ligand curdlan or with retinoic acid. Changes in oxygen consumption rate (OCR) **(A)** and extracellular acidification rate (ECAR) **(B)** over time upon injection of glucose, oligomycin, FCCP, and rotenone and antimycin A (Rot/AA) in naïve and IL-4 stimulated macrophages cultured in the presence of retinoic acid or curdlan. Based on these measurements, various metabolic parameters were determined as described in Supplementary Figure S2 (*n* = 3, ±SEM).
