# Supplementary figures and images for: Intestinal Macrophages Balance Inflammatory Expression Profiles via Vitamin A and Dectin-1-Mediated Signaling
Source: Front Immunol. 2020 Mar 31;11:551. doi: 10.3389/fimmu.2020.00551 (PMC7138104; doi:10.3389/fimmu.2020.00551)

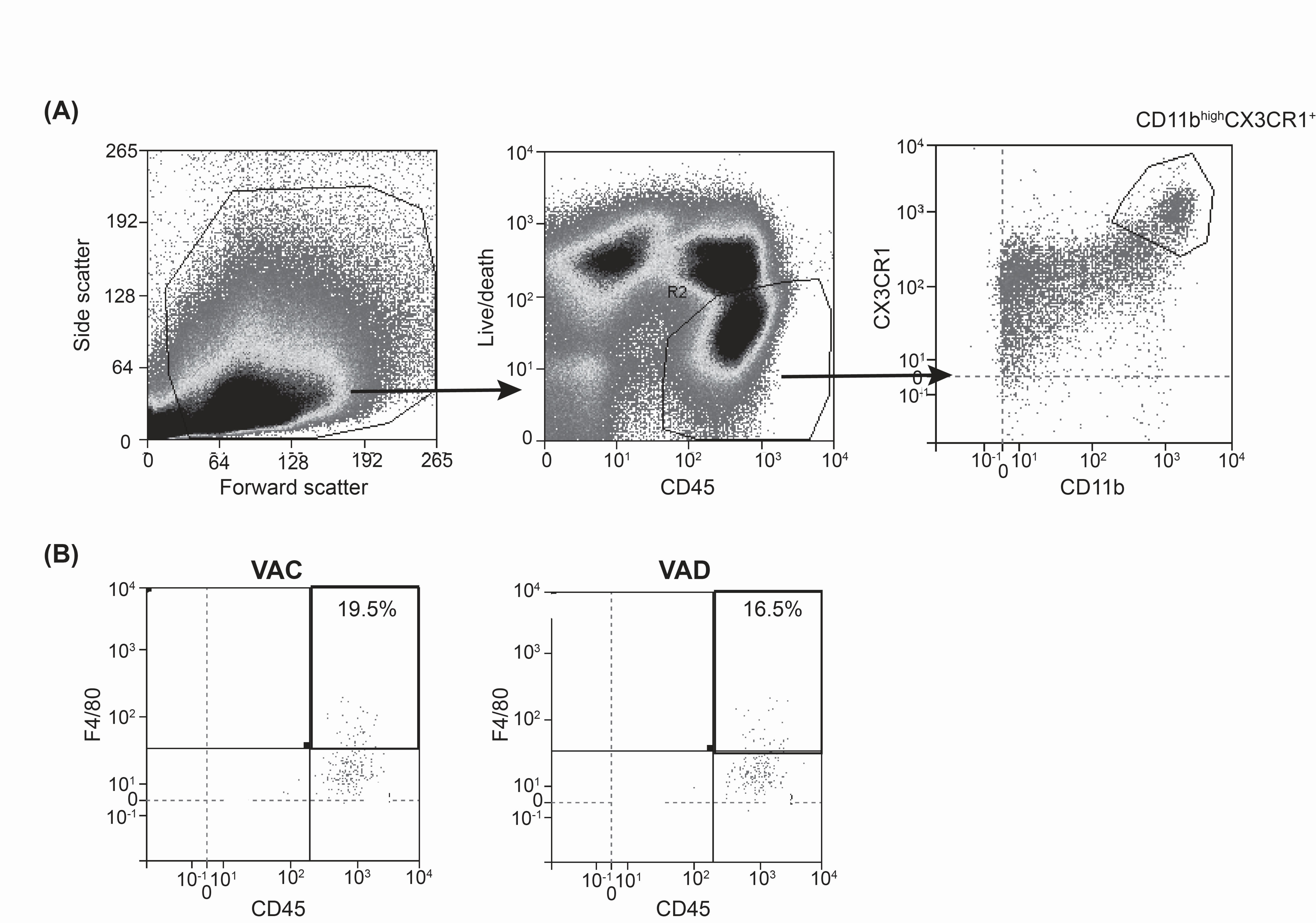

Supplement: FIGURE S1 — Gating strategy Small intestines were removed and made into a single cell suspension. (A) Live CD45+ cells were analyzed for CX3CR1 and CD11b expression and a gate for CD11bhighCX3CR1+ macrophages was set. (B) Gating strategy to determine proportion of F4/80+CD45+ cells of all lamina propria cells in vitamin A competent (VAC) and vitamin A deficient (VAD) mice. [file Image_1.JPEG]

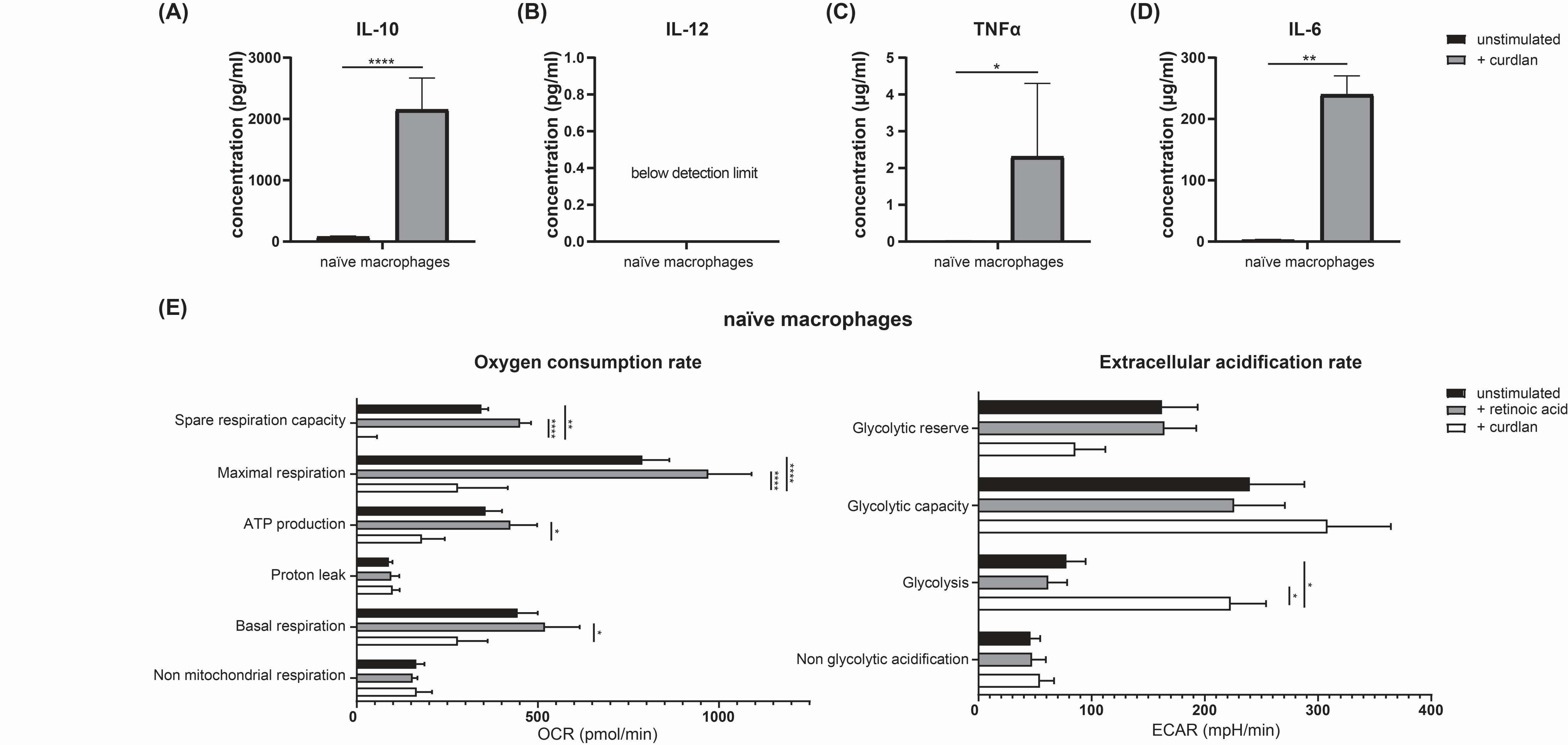

Supplement: FIGURE S2 — Effects of Dectin-1 stimulation in naïve macrophages. Bone marrow derived macrophages were cultured for 24 h with or without retinoic acid and were stimulated with the Dectin-1 ligand curdlan for an additional 24 h (A–D). Concentration of IL-10 (A), IL-12 (B), TNFα (C), and IL-6 (D) in the supernatants of cell cultures of IL-4 stimulated or LPS/IFNγ stimulated macrophages after stimulation with or without curdlan determined by ELISA. (E) Oxygen consumption rate (OCR) and extracellular acidification rate (ECAR) of naïve macrophages after stimulation with retinoic acid or curdlan. Various metabolic parameters were determined as described in Supplementary Figure S3. Changes in OCR and ECAR over time are represented in Supplementary Figure S4. Significant differences are indicated by ∗p < 0.05, ∗∗p < 0.01, ∗∗∗p < 0.005, or ****p < 0.001, (n = 3, ±SEM). [file Image_2.JPEG]

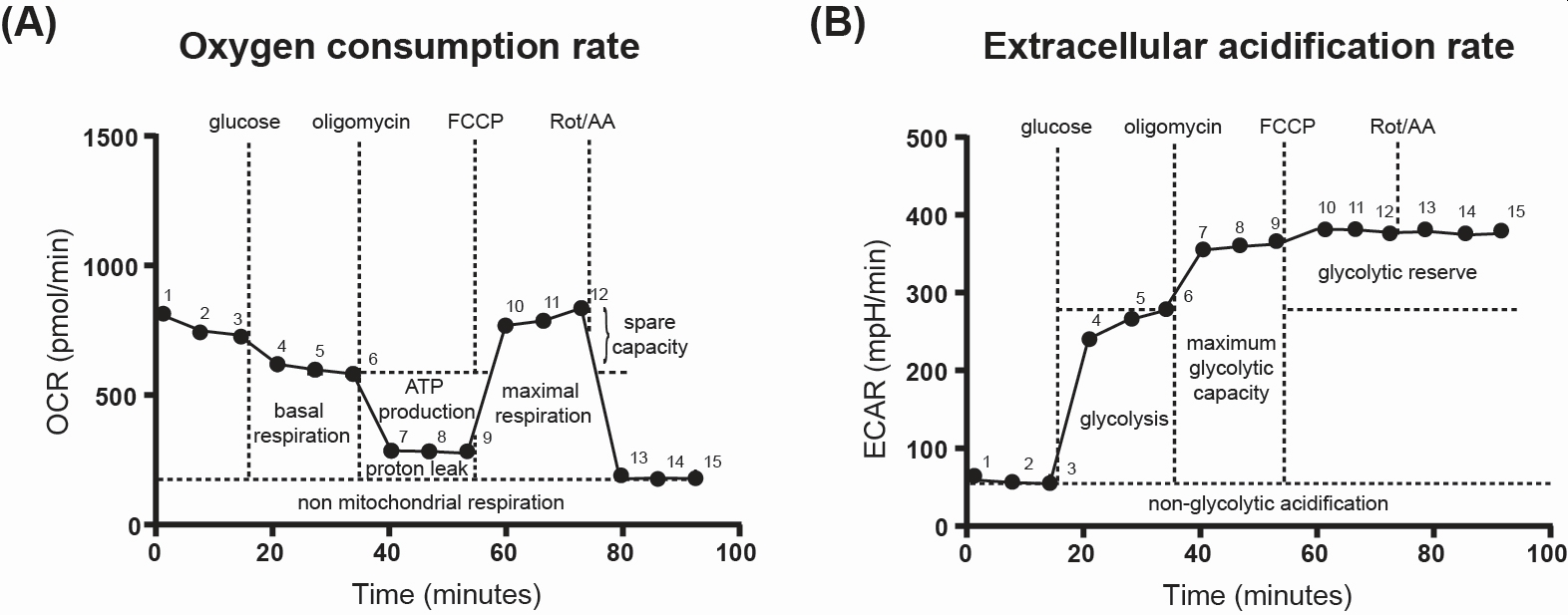

Supplement: FIGURE S3 — Analysis method extracellular flux assay. (A) When analyzing the oxygen consumption rates (OCR in pMoles/min), oligomycin injection allows to calculate the oxygen consumption used for mitochondrial ATP synthesis. Carbonyl cyanide 4-(trifluoromethoxy)phenylhydrazone (FCCP) uncouples mitochondrial respiration and the corresponding OCR measurements yield data about the maximal and spare respiratory capacity. Finally, injection of rotenone (Rot) and antimycin A (AA) block mitochondrial complex I and III and the residual OCR represents the non-mitochondrial oxygen consumption. (B) After the injection of glucose, the increase in extracellular acidification rate (ECAR in mpH/min) represents the glycolysis rate. The additional increase in ECAR after ATP synthase inhibition with oligomycin (OM) provides information about the glycolytic reserve and capacity. After normalization for cell numbers using protein concentration, following metabolic parameters are calculated as follows:Basal respiration = OCR (6) – OCR (15)Proton leak = OCR (9) – OCR (15). ATP production = OCR (6) – OCR (9). Maximum respiration = OCR (12) – OCR (15). Spare respiratory capacity = OCR (12) – OCR (6). Non-glycolytic acidification = ECAR (3). Glycolysis = ECAR (6) – ECAR (3). Maximum glycolytic capacity = ECAR (9) – ECAR (3). Glycolytic reserve = ECAR (9) – ECAR (6). This figure and legend were adapted from a previously published report (24). [file Image_3.JPEG]

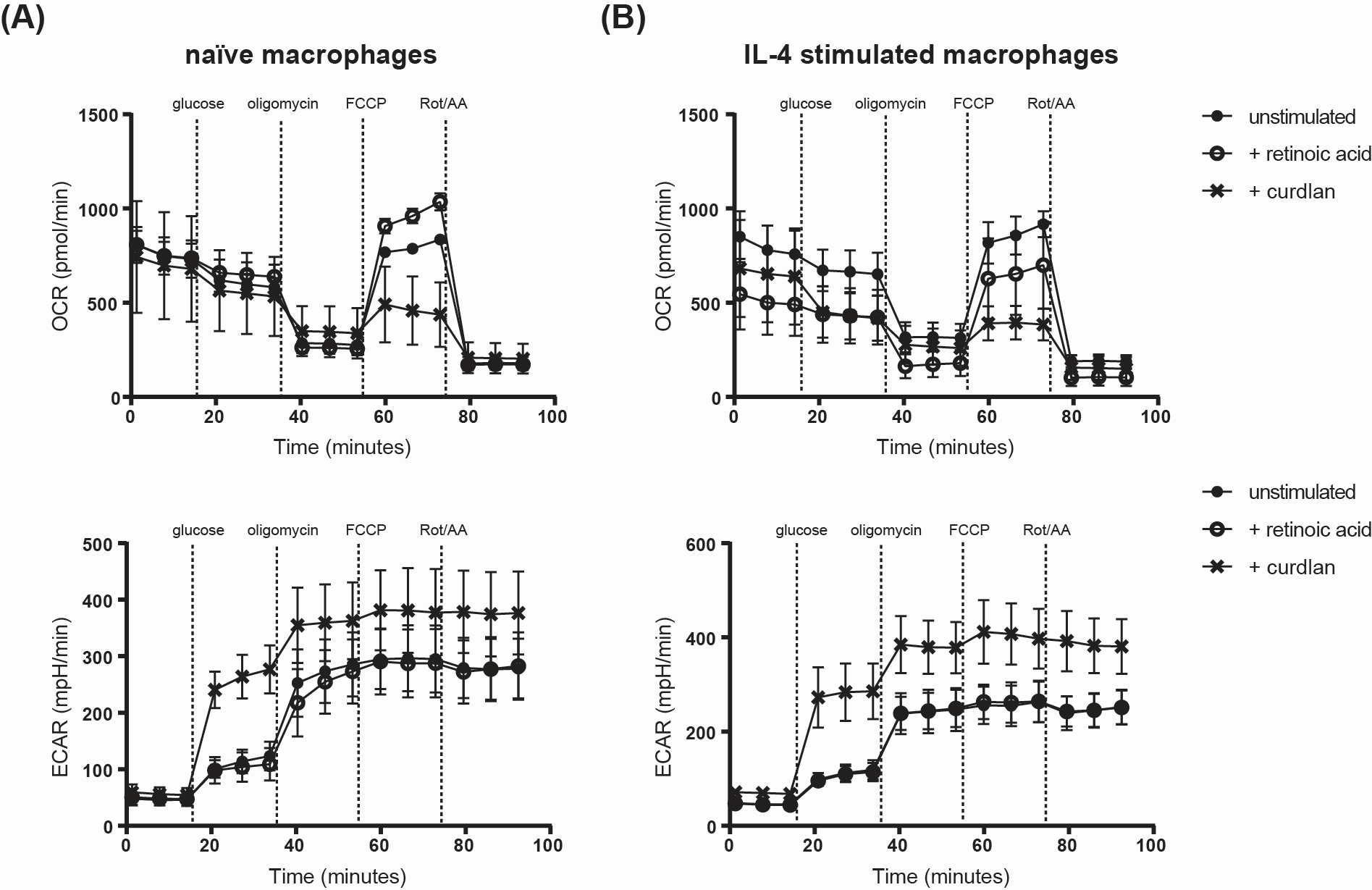

Supplement: FIGURE S4 — Changes in oxygen consumption rate and extracellular acidification rate of naive and IL-4 stimulated macrophages over time. Bone marrow derived macrophages were cultured and kept unstimulated or were skewed toward an anti-inflammatory phenotype using IL-4. Subsequently, macrophages were stimulated with the Dectin-1 ligand curdlan or with retinoic acid. Changes in oxygen consumption rate (OCR) (A) and extracellular acidification rate (ECAR) (B) over time upon injection of glucose, oligomycin, FCCP, and rotenone and antimycin A (Rot/AA) in naïve and IL-4 stimulated macrophages cultured in the presence of retinoic acid or curdlan. Based on these measurements, various metabolic parameters were determined as described in Supplementary Figure S2 (n = 3, ±SEM). [file Image_4.JPEG]
